# Study on the NO removal efficiency of the lignite pyrolysis coke catalyst by selective catalytic oxidation method

**DOI:** 10.1371/journal.pone.0182424

**Published:** 2017-08-09

**Authors:** Lei Zhang, Xin Wen, Zhenhua Ma, Lei Zhang, Xiangling Sha, Huibin He, Tianyou Zeng, Yusu Wang, Jihao Chen

**Affiliations:** 1 School of Geology and Environment, Xi’an University of Science and Technology, Xi’an, China; 2 China National Heavy Machinery Research Institute Co., Ltd, Xi’an, China; Universita degli Studi della Tuscia, ITALY

## Abstract

Selective catalytic oxidation (SCO) method is commonly used in wet denitration technology; NO after the catalytic oxidation can be removed with SO_2_ together by wet method. Among the SCO denitration catalysts, pyrolysis coke is favored by the advantages of low cost and high catalytic activity. In this paper, SCO method combined with pyrolysis coke catalyst was used to remove NO from flue gas. The effects of different SCO operating conditions and different pyrolysis coke catalyst made under different process conditions were studied. Besides, the specific surface area of the catalyst and functional groups were analyzed with surface area analyzer and Beohm titration. The results are: (1) The optimum operating conditions of SCO is as follows: the reaction temperature is 150°C and the oxygen content is 6%. (2) The optimum pyrolysis coke catalyst preparation processes are as follows: the pyrolysis final temperature is 750°C, and the heating rate is 44°C / min. (3) The characterization analysis can be obtained: In the denitration reaction, the basic functional groups and the phenolic hydroxyl groups of the catalyst play a major role while the specific surface area not.

## 1 Introduction

NO is an unwanted by-product of high temperature combustion where N_2_ from the air combines with O_2_ to form oxides of nitrogen [1]. Legislation requires the removal of these oxides from exhaust streams in most developed societies [2].At present, the denitration technology of selective catalytic reduction (SCR) has realized industrialization all over the world with the advantage of higher denitration efficiency and higher utilization among numerous flue gas denitration technologies [3]. However, the SCO method can not only remove NO separately after the dust-remover and desulfurization equipment but also can lead to simultaneous desulfurization and denitration [4–6]. So it is considered to have a great application foreground. Nowadays, the SCO catalyst types are mainly activated carbon, metallic oxide catalyst, noble metal catalyst, and molecular sieve based catalysts. The activity of activated carbon is high at low temperature, but it began to decline when the temperature is over 100°C [7–9]. The metal oxide catalyst is unstable and has low catalytic activity under high temperature condition; In addition, the SO_2_ and other substances present in the flue gas can reduce its activity or service life. The catalytic activity of the noble metal catalyst is higher than other catalysts in the catalytic reduction process, however, the consumption of the noble metal catalyst as the reducing agent is large, the operation cost is high, and sulfur poisoning and oxygen suppression are easy to occur in the selective catalytic oxidation process [10]; The activity of molecular sieve catalyst is high in the high temperature area, and the temperature range of high activity is wide, but the problems of water inhibition, sulfur poisoning and low temperature can limit its industrial application [11].

Rui Yao, Tao Yin has found that the water resistance and sulfur resistance of the pyrolysis coke catalyst, the results showed the co-addition of SO_2_ and H_2_O could decrease the catalytic activity and cause irreversible poisoning, however, pyrolysis coke is favored by the advantages of low cost and high catalytic activity in the SCO denitration catalyst, and is considered to be the most promising material for decomposing NO [12]; Zongbin Zhao, Wen Li’s research has showed that pyrolysis coke can act as a catalyst or catalyst carrier, and its activity was affected by temperature, pressure, specific surface area, pore volume and semi-coke alkali metal and alkaline earth metal content; On this basis, Shinichi Miura studied the effect of the final pyrolysis temperature on the specific surface area and pore structure, finding how the internal structure of the coal changed during the pyrolysis progress [13–17]; In addition, it is a better choice to use pyrolysis coke as the desulfurization and denitration catalyst, because it contains more material and entrance, and the hole will extend the residence time when the gas goes through the pyrolysis coke, and the catalyst itself contains metal ions which can be used as the catalyst, these performances make the pyrolysis coke as a better catalyst to remove NO from the gas[18].

In this study, the preparation process of different SCO operation process and pyrolysis coke catalyst was designed for the above deficiencies [19]. On this basis, the effects of different pyrolysis final temperature, different heating rate and different volatile conditions of the catalyst on NO removal efficiency were studied. The NO removal efficiency refers to the percent of the amount of NO removed during the denitrification process in the amount of NO in the raw gas. The functional groups and specific surface area were characterized by Boehm titration and BET specific surface area methods [20]. The main steps were as follows: (1) The effects of different SCO reaction temperature on NO removal efficiency were investigated and analyzed by fixing NO concentration and oxygen content; (2) The effects of different SCO oxygen content on NO removal efficiency were investigated and analyzed by fixing NO concentration and reaction tower temperature; (3) The effects of pyrolysis coke on the removal efficiency of NO were investigated and analyzed; (4) The effects of pyrolysis coke made by different heating rates on NO removal efficiency were investigated and characterized; (5) The effects of pyrolysis coke with different volatile content on the removal rate of NO were investigated and characterized [21].

## 2 Materials and methods

### 2.1 Material preparation

The raw material was Yi Min lignite. It was sieved with 30–40 mesh sieves after natural drying and grinding.

The proximate analysis of the lignite was: Before the distillation, the quality of the dry distillation tube was 97.411g; the quality of the cone bottle was 125.630g, the quality of coal samples was20.000g, and the total quality of dry distillation tube and coal sample was 117.409g. After the distillation, the total quality of dry distillation tube and half coke was 109.857g, the total quality of cone bottle, tar and water was 129.538g; The cumulative gas flow was 2.5L, the volume of distilled water was 3.7mL.So the reduced quality of coal samples was 7.552g(117.409g-109.857g),the quality of tar and water was 4.178g (129.538g–125.360g), and the quality of the gas was 3.2325g (2.5L×1.293g/L, assume that the gas density was air density, ρ = 1.293g/l, calculate the gas quality according the formula m = ρ×V). The total quality of the tar, water and gas was 7.4105g (4.178g+3.2325g), thus the acceptable deviation between the reduced quality of the coal samples was only 0.1415g(7.552g-7.4105g),that was 0.7%(0.1415g/20g).Above all, the volatile content of the Yimin lignite in this research was 37.76%(7.552g/20g),the water content was 18.5%(3.7g/20g).

### 2.2 Methods

**Fig 1:**
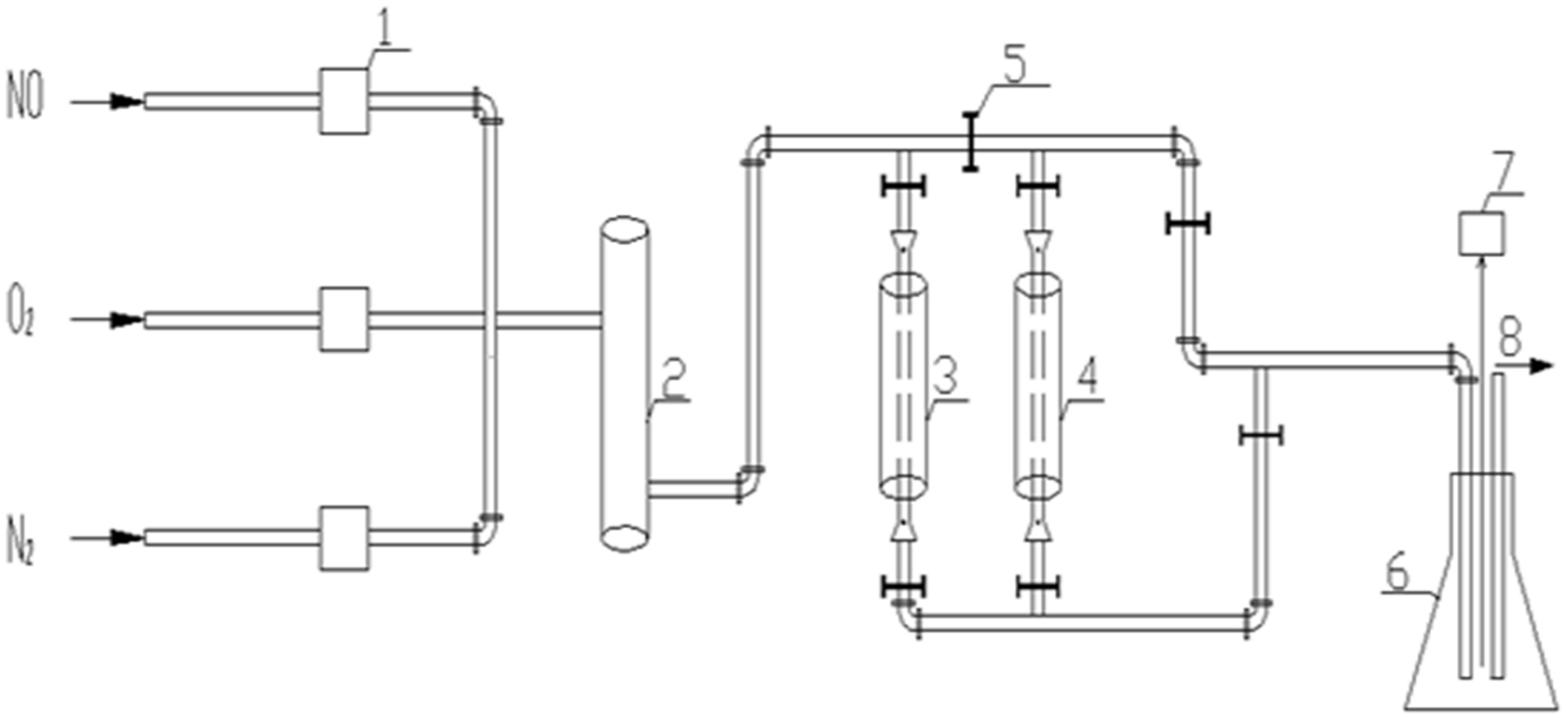
The schematic diagram used for experiment. The devices above are: 1-flowmeter, 2-mixing tank, 3-reaction tower A, 4-reaction tower B, 5-valve control, 6-testing cylinder, 7-flue gas analyzer, 8-exhaust gases.

The schematic diagram used for this study is shown as followed (Fig 1).

At first, preparing the catalyst by raw lignite materials pyrolysis coking, the pyrolysis coke can be used as flue gas denitration catalyst, with the device above, determining its denitration efficiency, and then evaluating the catalyst activity.

NO in the flue gas emitted by the plant can react with O_2_ under heating conditions to produce NO_2_. In the study, using mixture gas of NO, O_2_ and N_2_ to simulate the flue gas, the three gases get mixed in the mixing tank, and then undergoing denitration reaction in the reaction tower A and B, detecting the NO content with the flue gas analyzer after the reaction to determine the removal efficiency [22].

When there is no catalyst in the reactor, the optimum SCO operating conditions can be determined by changing the concentration of O_2_ in the feed gas and the temperature of the reaction column. When the catalyst is placed in the reactor, the denitration effect of the catalyst is determined by this apparatus [23].

### 2.3 Catalyst preparation

1. Prepare the pyrolysis coke catalyst at different pyrolysis final temperatures and different heating rates: The lignite particles were placed in a quartz tube after sieving and inserted into an electric furnace, set the final pyrolysis temperature at 550°C, 650°C, 750°C and 850°C respectively, heat the lignite with the heating rate 12°C/min, 22°C/min and 44°C/min respectively, calculate and set up the time, the heating lasts for about 1 hour until it reaches the final pyrolysis temperature, stop heating when tature stabilized [24], then seal and store the coke after cooling.
2. Determine the volatile of the pyrolysis coke catalyst. Determine the volatile of all the 12 catalysts (four final pyrolysis temperatures with three heating rates). The volatile contents of the catalysts whose heating rate is 44°C/min and whose final pyrolysis temperature is 750°C are shown as follows (Table 1).

**Table 1:** The volatile of the pyrolysis coke catalyst prepared with different conditions.

Sample	Volatile	Sample	volatile
550°C coke (fresh)	11.5%	12°C/min coke (fresh)	8.2%
650°C coke (fresh)	9.7%	20°C/min coke (fresh)	8.7%
750°C coke (fresh)	8.0%	44°C/min coke (fresh)	5.0%
850°C coke (fresh)	3.7%		

**Notes**: 850°C coke refers to the pyrolysis coke whose final pyrolysis temperature is 850°C, the same as the 550°C coke,650°C coke,750°C coke;44°C/min coke refers to the pyrolysis coke whose heating rate is 44°C/min, the same as the 12°C/min coke, 20°C/min coke.

### 2.4 Catalyst activity evaluation

The catalyst activity was evaluated in the simulated flue gas plant (Fig 1). The simulated flue gas flow: G = 1L/min, the composition of the flue gas after mixing: NO (18ml/min), O_2_ (60 ml/min), N_2_ (922ml/min) [25]. The volume fraction of NO was analyzed by a gas detector (Testo 340). Usually, we believe that the catalyst with higher denitration efficiency also has a better activity.

### 2.5 Catalyst characterization method

The pyrolysis coke catalyst was characterized by Boehm titration to determine the content of the function groups. The specific surface area was measured by surface area analyzer 3H-2000A.

## 3 Results and discussion

### 3.1 The influence of different operating conditions on NO removal efficiency

#### 3.1.1 The influence of SCO temperature on NO removal efficiency

**Fig 2:**
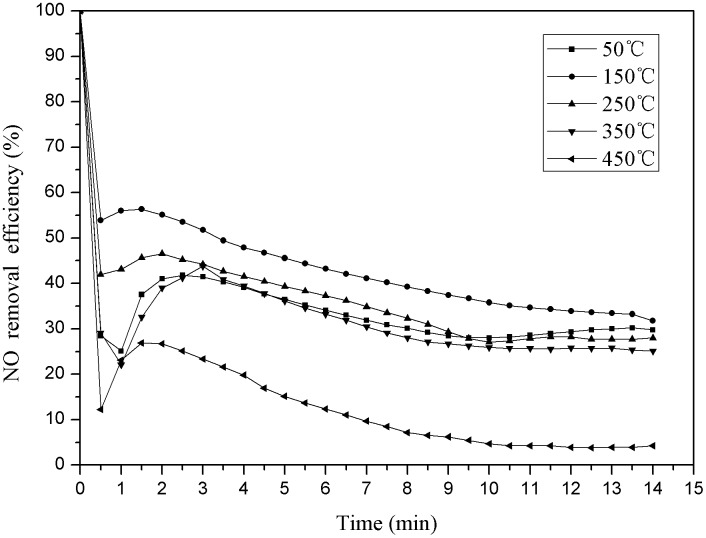
Denitration efficiency under different SCO temperature conditions. Notes: when the time is 0, we cannot detect any NO in the exhaust gas, so we suppose the denitration efficiency is 100% when the time is 0, therefore, the data trend should be determined from the first observation point “0.5min” position actually. This treatment is also applied for the following figures.

This experiment was carried out without any catalyst, the temperature of the reaction tower was set at 50°C, 150°C, 250°C, 350°C and 450°C respectively, the oxygen volume fraction was 6%. The effect of temperature on NO removal rate was researched. The results are shown as follows (Fig 2).

As shown above (Fig 2), the denitration efficiency shows a tendency to increase first and then decrease with the reaction time increasing. The denitration efficiency is 41% when the temperature is 50°C, and the highest denitration efficiency is 55% when the temperature is 150°C. It showed that the optimum temperature should be between 50°C and 350°C in the denitration process [26]. The conversion process of NO to NO_2_ is more likely to occur at low temperature because the NO oxidation reaction is an exothermic reaction. It is not conducive to the formation of NO_2_, or the reaction is inhibited when the temperature exceed 350°C, NO converts to more NO_2_ when the temperature is below 200°C, and NO_2_ is unstable and decomposes into NO slowly when the temperature exceed 200°C. More NO_2_ will be decomposed and the removal rate of NO will decrease with the temperature increasing [27]. When the temperature reaches 150°C, the reaction of NO and O_2_ generates more NO_2_, and the denitration efficiency is the best.

#### 3.1.2 The influence of oxygen content on NO removal efficiency

**Fig 3:**
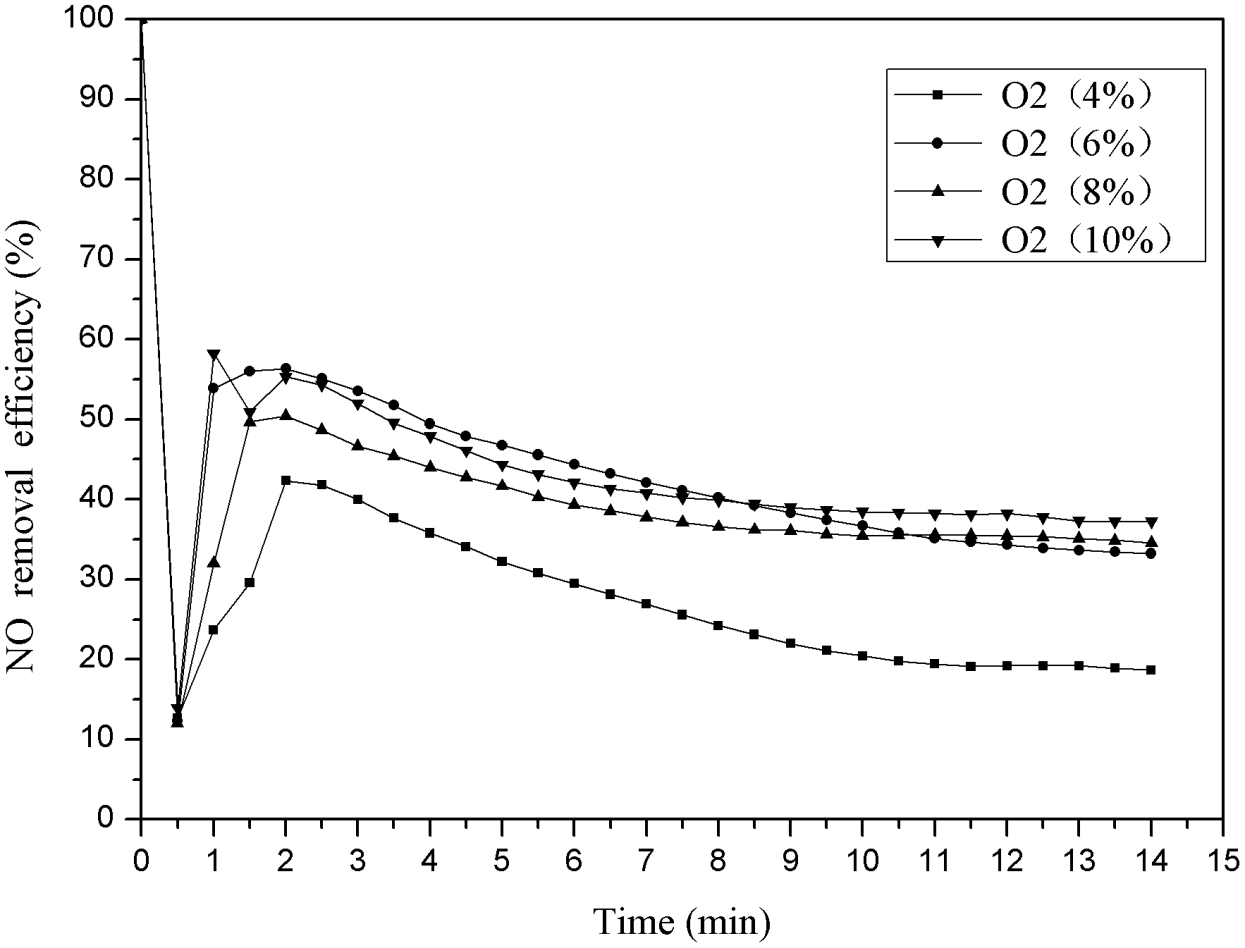
Denitration efficiency under different oxygen content.

This experiment was carried out without any catalyst, the oxygen volume fraction was 4%, 6%, 8%, and 10% respectively, the reaction temperature was 150°C. The influence of oxygen content on the denitration efficiency was researched. The results were shown as follows (Fig 3).

As shown in Fig 3, the denitration efficiency shows a tendency to increase first and then stabilize with the reaction time increasing. The denitration efficiency is the lowest when the oxygen concentration is 4%, the denitration efficiency is the best when the oxygen concentration is 6%; the denitration efficiency under the condition of the 8% oxygen concentration is just below the denitration efficiency under the condition of the 6% oxygen concentration, the trend of denitration efficiency of the 10% oxygen concentration is almost the same as the efficiency when the oxygen concentration is 6%. From this, the denitration efficiency is essentially constant with the oxygen concentration increasing when the oxygen concentration and NO concentration have reached a certain ratio [28–30]. The reason is that the collision probability of NO and O_2_ increase with the oxygen content increasing from 4% to 6%, thus the denitration efficiency increase. The reaction has been almost saturated when the O_2_ concentration reaches 8%, so the denitration efficiency is essentially constant because the concentration of NO is the limiting factor [31].

### 3.2 The influence of the pyrolysis coke catalyst on NO removal efficiency

#### 3.2.1 The effect of the pyrolysis coke catalyst on the adsorption of NO

**Fig 4:**
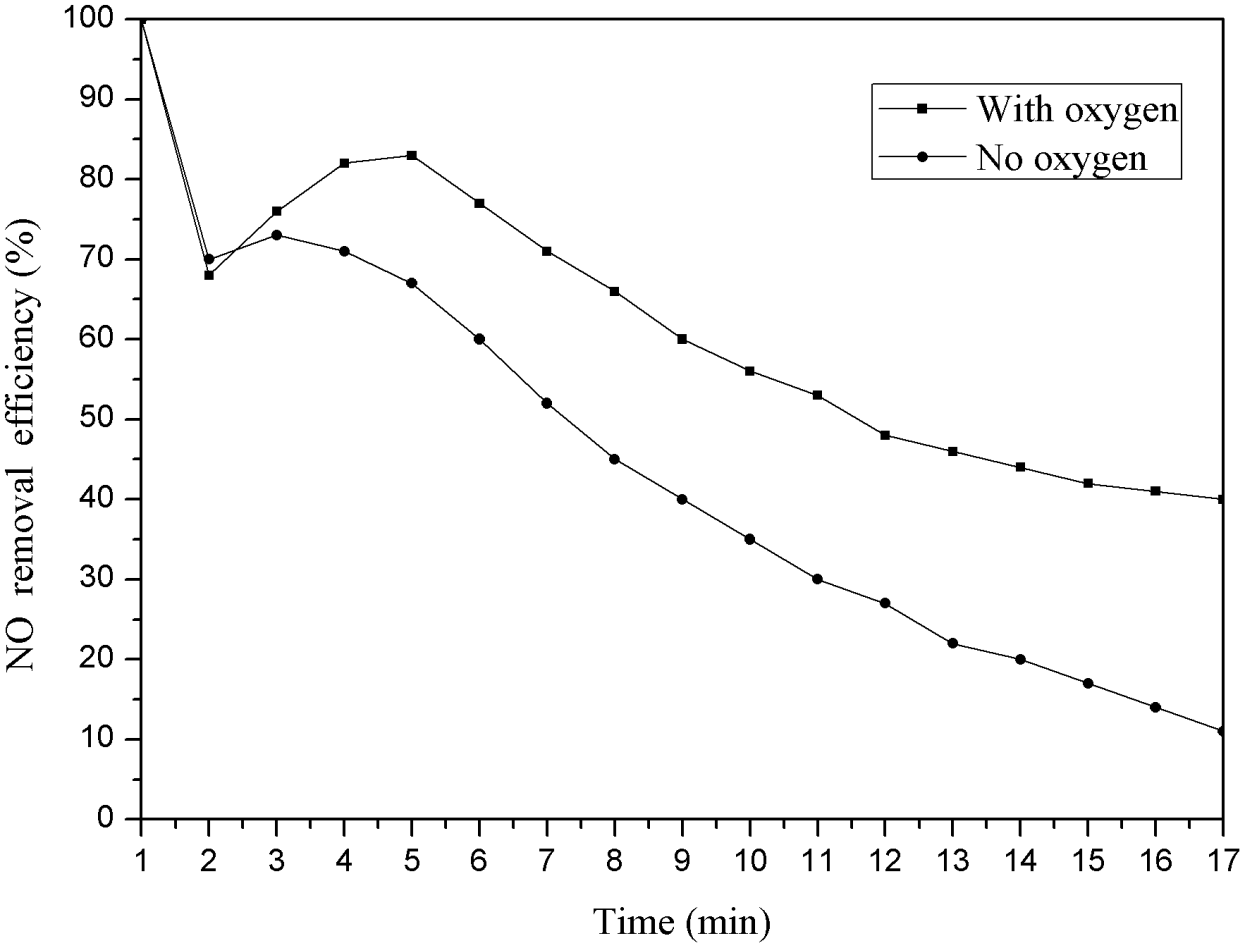
Denitration efficiency of pyrolysis coke catalyst with or without O_2_.

Weighing 3g pyrolysis coke catalyst whose pyrolysis final temperature was 750°C in this experiment. Put it into the temperature control tower and set the temperature to 150°C, the following reaction was carried out with the O_2_ was introduced (the concentration of oxygen is 6%) and the O_2_ was not introduced, the results were as follows (Fig 4).

As shown in Fig 4, the pyrolysis coke catalyst only make the adsorption under the condition of no O_2_, the pyrolysis coke catalyst has catalytic oxidation effect when the reaction temperature is 150°C under the condition of putting into O_2_, and the denitration efficiency is over 40% when the reaction time reaches 17 min. Based on the analysis and comparison, the adsorption effect can be neglected in the experiment due to the adsorption capacity of pyrolysis coke catalyst is lower. It is found that the reduced NO is converted into NO_2_ completely [32].

#### 3.2.2 The influence of the different final temperature pyrolysis coke catalyst on NO removal efficiency

**Fig 5:**
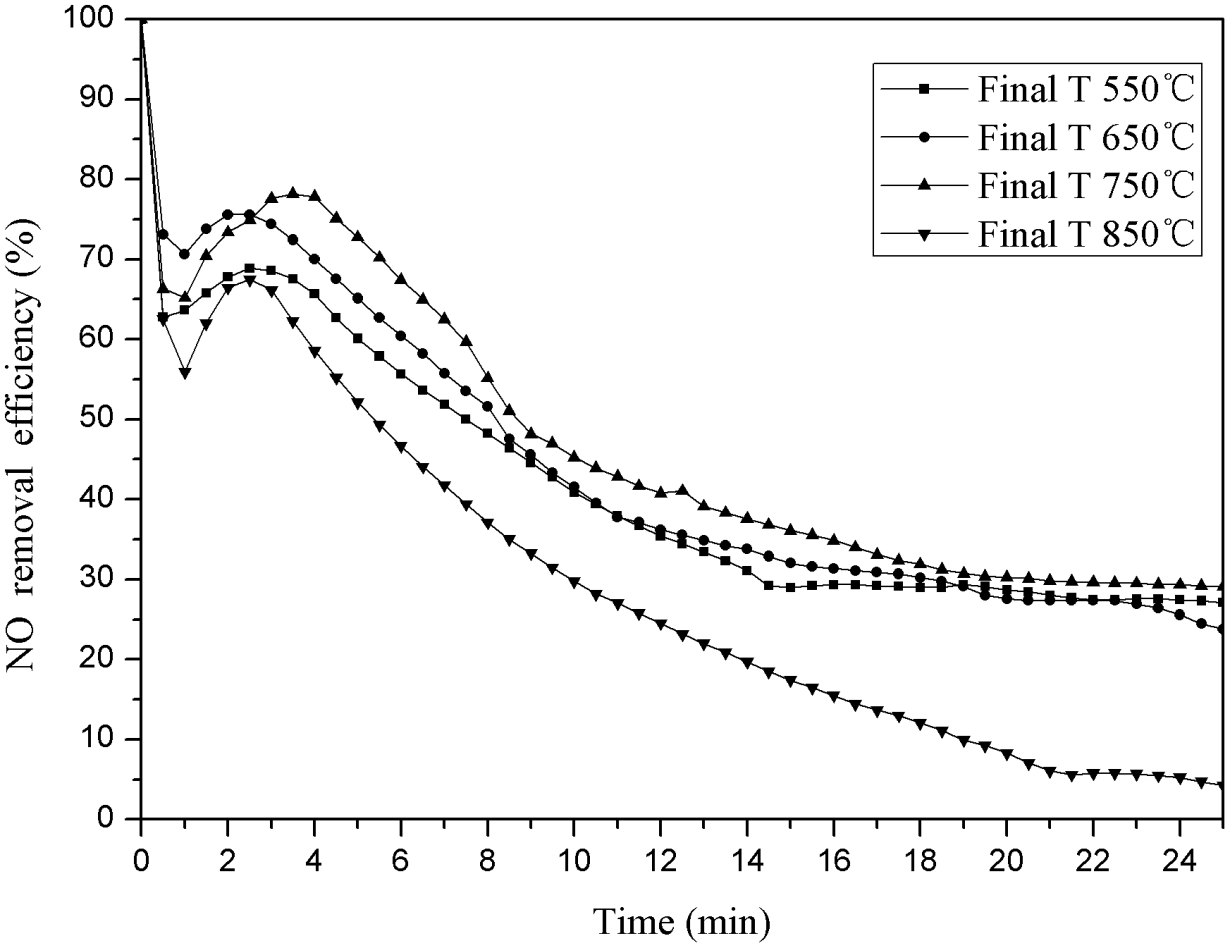
Denitration efficiency for different final temperature pyrolysis.

Weighing 3g pyrolysis coke catalyst whose heating rate was 44°C/min and final catalyst temperatures was 550°C, 650°C, 750°C and 850°C respectively. The reaction was carried out in a reaction tower at 150°C and 6% oxygen concentration, the results were as follows (Fig 5).

As shown in Fig 5, the denitration efficiency of the pyrolysis coke catalyst increases first and then decreases with the pyrolysis final temperature increasing, the denitration efficiency is the best when the pyrolysis final temperature of the pyrolysis coke catalyst is 750°C.

#### 3.2.3 The effect of the different heating rate pyrolysis coke catalyst on the NO removal efficiency

**Fig 6:**
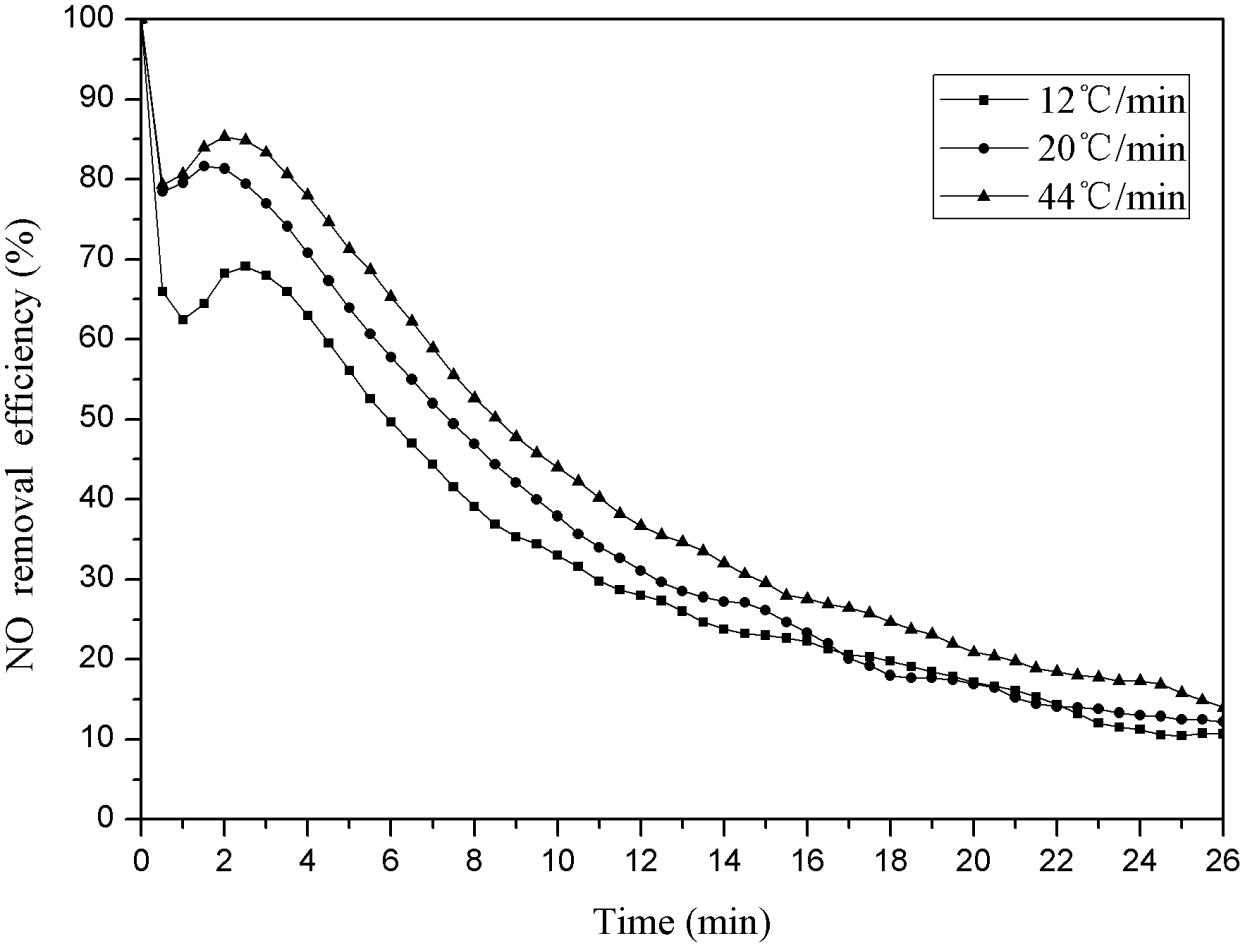
Denitration efficiency of pyrolysis coke with different heating rates.

Weighing 3g pyrolysis coke catalyst respectively whose pyrolysis final temperature was 750°C and heating rate was 12°C/min, 20°C/min and 44°C/min respectively. The reaction was carried out in a reaction tower at a temperature of 150°C and an oxygen concentration of 6%. The results were as shown below (Fig 6).

As shown in the Fig 6, the denitration efficiency of the pyrolysis coke catalyst shows the increasing trend with the heating rate increasing. According to the result (Fig 6), the denitration efficiency is the best and the effective catalysis time is the longest when the heating rate is 44°C/min.

#### 3.2.4 The influence of the different volatile pyrolysis coke catalyst on the NO removal efficiency

**Fig 7:**
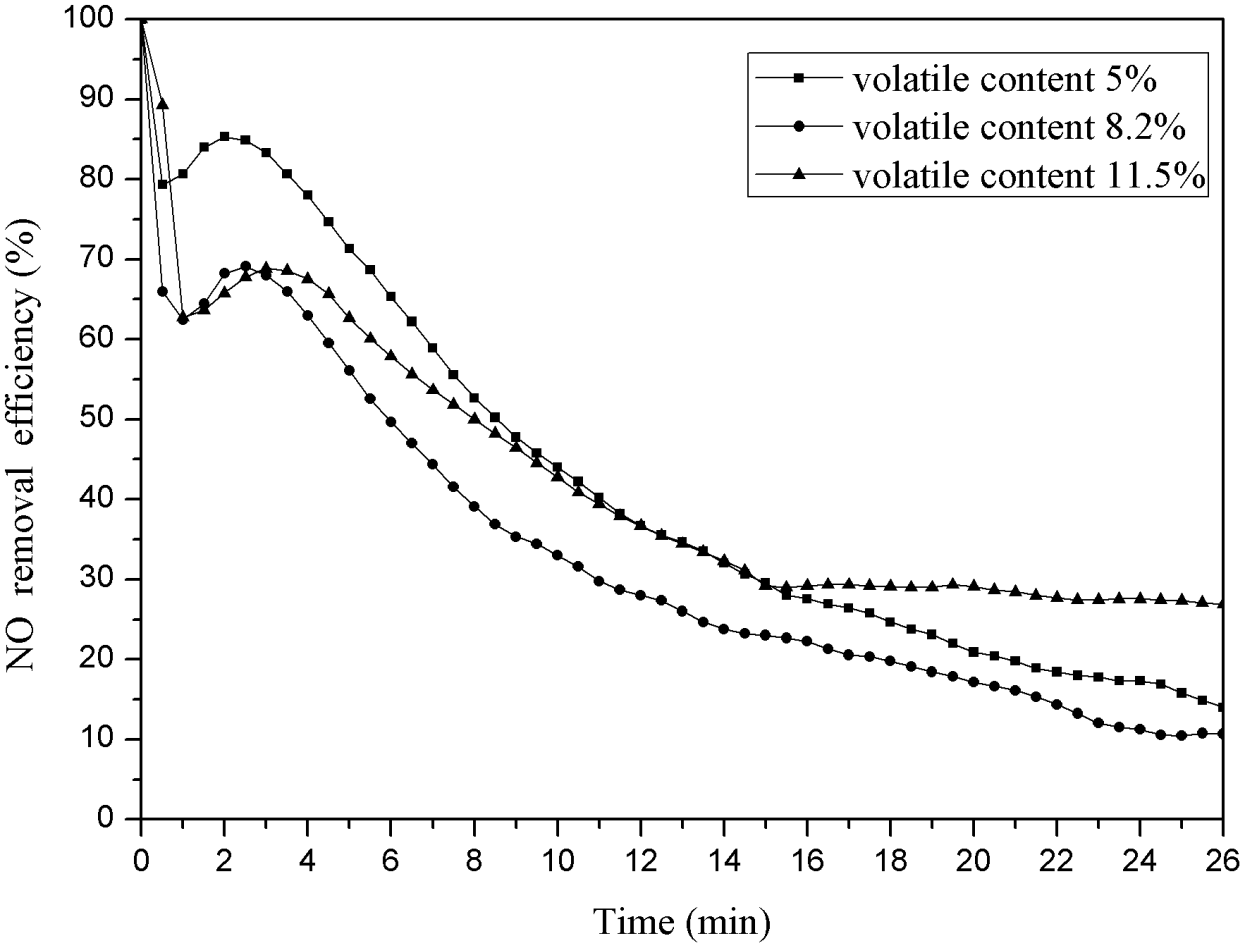
Denitration efficiency of pyrolysis coke with different volatiles.

Weighing 3g pyrolysis coke catalyst whose final pyrolysis temperature were 750°C and the volatile content was 5%, 8.2%, 11.5% respectively (the pyrolysis coke catalyst volatile content has been measured and marked before the experiment). The reaction was carried out in a reaction tower at a temperature of 150°C and an oxygen concentration of 6%; the results were as shown below (Fig 7).

As shown in Fig 7 the denitration efficiency of the pyrolysis coke catalyst decreases first and then increase with the volatile content increasing. The low volatile content is due to high temperature roasting caused by coal metal ions and organic matter escape [33]. The metal ions and organic matter plays an important role in the denitration, thus reducing its denitration rate. The pyrolysis coke catalyst volatile is higher when the final temperature is 450°C because the low-temperature roasting do not change the coal performance, and the combustible material is not burned completely [34].

The denitration efficiency is best when the pyrolysis coke catalyst volatile is 5% because its specific area is in a certain range, the distribution of the oxygenic functional group is uniform on the surface, the activity sites are more and the multihole carbon skeletons structure on the surface is well, so it is beneficial for adsorbing NO and occurring oxidation reactions, thereby the catalysis performance of the pyrolysis coke catalyst is better [35]. Meanwhile, the pyrolysis coke catalyst whose volatile is 11.5% contains more metal ions and organic matter, so the denitration efficiency is higher than that when the pyrolysis coke catalyst volatile is 8% after 15 min. So it can be concluded that the denitration efficiency of the pyrolysis coke catalyst will be better when the volatile is controlled in the certain range [36].

### 3.3 The functional groups characterization of fresh catalyst and post-reaction catalyst

Determine the surface functional groups of the fresh pyrolysis coke catalyst and the post-reaction pyrolysis coke catalyst by Boehm Titration. These catalysts were prepared in different pyrolysis final temperature with the same heating rate 44°C/min, the post-reaction catalyst has taken reaction at 150°C, O_2_ 6%; The result is shown as follows (Table 2).

**Table 2:** Functional groups of pyrolysis coke catalysts.

Sample	Acidic functional groups	Basic functional groups	Phenolic hydroxyl group	Carboxyl
550°C coke (fresh)	0.0000	2.0000	0.0103	0.0037
650°C coke (fresh)	0.0000	2.0500	0.0117	0.0033
750°C coke (fresh)	0.0000	2.1000	0.0113	0.0038
850°C coke (fresh)	0.0000	2.5500	0.0085	0.0045
550°C coke (reaction)	2.1500	0.0000	0.0000	0.0048
650°C coke (reaction)	2.0500	0.0000	0.0000	0.0050
750°C coke (reaction)	2.0000	0.0000	0.0000	0.0043
850°C coke (reaction)	2.5500	0.0000	0.0000	0.0037

Unit: mmol/g

As shown in the Table 2, the content of functional groups on the pyrolysis coke catalyst surface prepared with different pyrolysis final temperatures is different. The surface contents of the acidic functional groups are zero, while the basic functional groups gradually increase, and the content of phenolic hydroxyl groups and carboxyl groups has no significant difference with the temperature increasing. After reaction, the content of basic functional groups and phenolic hydroxyl groups all become zero and the content of acidic functional groups increase significantly. It can be seen from the result, the basic functional groups and phenolic hydroxyl groups play an important role in denitration reaction [37].

We have come to the conclusion from Fig 5 that the pyrolysis coke catalyst prepared in 750°C has the best denitration efficiency compare with the pyrolysis coke catalyst prepared in 550°C and 650°C, and as shown above (Table 2), the number of basic functional groups are increasing with the temperature increasing, it indicates that the basic functional groups has a great effect on the denitration efficiency. According to the Table 2, the content of the basic functional groups is the highest when the pyrolysis coke prepared in 850°C, but its denitration efficiency is the lowest, and the content of pyrolysis phenolic hydroxyl group in 850°C is the lowest. Therefore, it can be speculated that there is a synergetic relationship between the basic functional groups and the phenolic hydroxyl groups, and the pyrolysis coke catalyst has a better denitration performance when the two groups’ ratio is within a certain range [38–40].As the Table 3 shown, the surface functional groups of fresh and after- reaction pyrolysis coke catalyst prepared at the different heating rates.

**Table 3:** Boehm titrations of pyrolysis coke catalysts prepared at different heating rates.

sample	Acidic functional groups	Basic functional groups	Phenolic hydroxyl group	carboxyl
12°C/min coke (fresh)	0.0000	2.0500	0.0138	0.0033
20°C/min coke (fresh)	0.0000	2.0500	0.0110	0.0037
44°C/min coke (fresh)	0.0000	1.9000	0.0147	0.0038
12°C/min coke (reaction)	2.2	0.0000	0.0000	0.0050
20°C/min coke (reaction)	2.15	0.0000	0.0000	0.0037
**44°C/min coke (reaction)**	**2.05**	**0.0000**	**0.0000**	**0.0048**

Unit: mmol/g; The final pyrolysis temperature of these catalysts were 750°C; The reaction was carried out at 150°C, O_2_ 6%.

As can be seen from Table 3, the content of basic functional groups and phenolic hydroxyl functional groups become zero after the reaction, it indicates that the basic functional groups and phenolic hydroxyl functional groups plays a main role in the reaction progress [41]. But there is no obvious normal correspondence between the two functional groups and the heating rates; but according to the Table 2, the basic functional groups and the phenolic hydroxyl groups has a main role in promoting each other. The pyrolysis coke catalyst has a better denitration performance only when its proportion is within a certain range. It can be seen from the Fig 6, the denitration efficiency of the catalyst is the best when the heating rate is 44°C/min, and the volatilization of pyrolysis coke catalyst is lower. Therefore, the pyrolysis coke catalyst prepared at a fast heating rate has a better carbon structure and functional group distribution, so the denitration efficiency of the pyrolysis coke catalyst is better, too.

### 3.4 Specific surface area characterization of the fresh and the post-reaction pyrolysis coke catalyst

The specific surface area of the pyrolysis coke catalyst before and after the reaction was measured by surface area analyzer 3H-2000A, and the result was shown as follows (Table 4).

**Table 4:** Specific surface area of different pyrolysis final temperatures catalysts.

Sample	Specific surface area	Sample	Specific surface area
550°C coke (fresh)	42.1044	550°C coke (reaction)	39.5448
650°C coke (fresh)	51.3608	650°C coke (reaction)	37.8511
750°C coke (fresh)	129.649	750°C coke (reaction)	83.3659
850°C coke (fresh)	297.943	850°C coke (reaction)	291.187

Unit: m^2^/g; The heating rate of these catalyst was 44°C/min;The reaction was carried out at 150°C, O_2_ 6%.

From Table 4, it can be seen that the specific surface area of the catalyst increase with the reaction final temperature increasing, and the specific surface area of the catalyst decrease after the denitration, but the trend of the decreasing range is not obvious. The differences of the specific surface area increase first and then decrease with the pyrolysis coke final temperature increasing before and after reaction. This is because the structure change of the pyrolysis coke catalyst is small at the low temperature. Due to less active sites, the extent of the structural damage is low when the active sites are inactivated in the denitration process, so the change of the specific surface area is small [42]. The structure of pyrolysis coke catalyst gradually changes to form an active carbon framework with the pyrolysis final temperature increasing, and it has more active sites. The structure of the active site is damaged seriously, the pore size become larger and the specific surface area decrease rapidly after the pyrolysis coke catalyst deactivation when the active site participate in denitration reaction. A stable carbon framework is formed when the pyrolysis final temperature is raised to 850°C continuously, and the original active sites on the surface are stable and deactivate due to the high temperature. Therefore, the decrease of the specific surface area is not obvious after the denitration [43].

As shown in the Table 2 and Fig 5, there is more basic functional groups and phenolic hydroxyl groups on the pyrolysis coke catalyst surface and the denitration efficiency is the best when the pyrolysis final temperature is 750°C. It can be speculated, temperature and oxygen are secondary influence factors when the pyrolysis coke catalyst is under a denitration test in a reaction tower at 150°C, NO and thermal decomposition play the main role on the pyrolysis coke catalyst surface: making the surface change from alkaline to acidic. At the same time, the carbon structure is destroyed, the skeleton structure is collapsed, and the active site is covered to form the macroporous structure, leading to the denitration efficiency decreasing. The pyrolysis coke catalyst prepared at 750°C is relatively stable and not easily destroyed, so it has a good denitration effect in the denitration process[44–46].The specific surface areas of the pyrolysis coke catalyst prepared at different heating rates are shown in Table 5:

**Table 5:** Specific surface area of catalysts prepared at different heating rates.

sample	Specific surface area	sample	Specific surface area
12°C/min coke (fresh)	34.7361	12°C/min coke (reaction)	76.3397
20°C/min coke (fresh)	12.5834	20°C/min coke (reaction)	29.8775
44°C/min coke (fresh)	113.582	44°C/min coke (reaction)	112.288

Unit: m^2^/g. The final pyrolysis temperature of these catalyst was 750°C. The reaction was carried out at 150°C, O_2_ 6%

It can be seen from Table 5 that the specific surface area of the pyrolysis coke catalyst prepared with different heating rates has a large gap. As shown in the Fig 6 and Table 3, the denitration efficiency is the best when the heating rate is 44°C/ min. The functional groups on the surface can be distributed evenly on the pyrolysis coke catalyst surface, so that it has a good pore structure and uniform active distribution sites when the specific surface area is larger [47–48]. It can also be seen from the table, the increasing trend of the specific surface area is not significant when the heating rate is 44°C/ min after the denitration progress, and this is because the pyrolysis coke catalyst has formed more surface activity sites in a short time, and it does not be destroyed with the faster heating rate [49]. The pyrolysis coke catalyst reaction is slow in a short time when the heating rate was low, and the structure of pyrolysis coke catalyst is difficult to change. The rising temperature could change the pyrolysis coke catalyst surface structure to form the active sites, but the process is slow, the generated active sites has been destroyed and finally retains less, and the structure is instability. The air flow continues to impact the unstable structures form a new pore structure again, so the specific area increases [50–51]. Besides, as shown in the Table 4 and the Fig 5, the specific surface area of the pyrolysis coke catalyst prepared at 850°C is the biggest, but its denitration efficiency is not the best, and this has proved that the specific surface area is not the main factor to determine the denitration efficiency [52].

## 4 Conclusion

1. The denitration efficiency increases first and then decreases with the temperature increasing, and the denitration efficiency is the best under 150°C in the SCO process; The denitration efficiency increases and then maintains a steady state with the oxygen concentration increasing; the denitration efficiency is the best when the oxygen content is 6%.
2. The denitration efficiency of the pyrolysis coke catalyst increases first and then decreases with the pyrolysis final temperature increasing, the denitration efficiency is the best when the final pyrolysis temperature is 750°C. The denitration efficiency of pyrolysis coke catalyst increases with the heating rate increasing, the denitration efficiency of the pyrolysis coke catalyst is the best when the heating rate is 44°C/min. The denitration efficiency of pyrolysis coke catalyst tends to increase with the pyrolysis coke catalyst volatile decrease; the denitration efficiency is the best when the pyrolysis coke volatile is 5%.
3. Through the functional groups and specific surface area characterization, we learn that the basic functional groups and the phenolic hydroxyl groups of the catalyst plays a major role and promotes each other in the denitration reaction and that the specific surface area of the catalyst is not the main factor to determine the denitration efficiency.

## Supporting information

S1 File Experimental data. (XLSX)
